# Lessons From 20 Years of Studies of Wheat Genotypes in Multiple Environments and Under Contrasting Production Systems

**DOI:** 10.3389/fpls.2019.01745

**Published:** 2020-01-28

**Authors:** Juan M. Herrera, Lilia Levy Häner, Fabio Mascher, Jürg Hiltbrunner, Dario Fossati, Cécile Brabant, Raphaël Charles, Didier Pellet

**Affiliations:** ^1^ Varieties and Production Techniques, Plants and Plant Products, Agroscope, Nyon, Switzerland; ^2^ Field-Crop Breeding and Genetic Resources, Plant Breeding, Agroscope, Nyon, Switzerland; ^3^ Team Suisse Romande, FiBL, Research Institute of Organic Agriculture, Lausanne, Switzerland

**Keywords:** grain yield, protein yield, germplasm, variety, crop management, plant breeding, cropping systems

## Abstract

Identifying opportunities and limitations for closing yield gaps is essential for setting right the efforts dedicated to improve germplasm and agronomic practices. This study analyses genotypes × environments interaction (G × E), genetic progress, and grain yield stability under contrasting production systems. For this, we analyzed datasets obtained from three Swiss trial-networks of winter wheat that were designed to evaluate genotypes under organic farming conditions, conventional management with low-inputs (150 kg nitrogen (N) ha^−1^ with no fungicide application) and conventional management with high-inputs (170 kg N ha^−1^ with fungicide application). The datasets covered the periods from 1998 to 2018 for organic and conventional management with low-inputs and from 2008 to 2018 for conventional management with high-inputs. The trial-networks evaluated each year an average of 36 winter wheat genotypes that included released varieties, advanced breeding lines, and lines for registration and post-registration in Switzerland. We investigated within each trial-network the influence of years, genotypes, environments and their interactions on the total variance in grain yield and grain N concentration using variance components analyses. We further applied mixed models with regression features to dissect genetic components due to breeding efforts from non-genetic components. The genotype as a single factor or as a factor interacting with the environment or the year (G × E, G × year, and G × E × year) explained 13% (organic), 20% (conventional low-inputs), and 24% (conventional high-inputs) of the variance in grain yield, while the corresponding values for grain N concentration were 29%, 25%, and 32%. Grain yield has stagnated since 1990 for conventional systems while the trend under organic management was slightly negative. The dissection of a genetic component from the grain yield trends under conventional management showed that genetic improvements contributed with 0.58 and 0.68 t ha^−1^ y^−1^ with low- and high- inputs, respectively. In contrast, a significant genetic source in the grain yield trend under organic management was not detected. Therefore, breeding efforts have been less effective on the wheat productivity for organic farming conditions than for conventional ones.

## Introduction

In Switzerland, bread wheat is the most cultivated crop with a cultivated area of 75,830 ha in 2018. This area corresponds to 18% of the arable land and yielded 412,000 tons of grains in 2017 (Swiss Federal Statistical Office, 2018). From the area cultivated with cereals, 7.6% was cultivated under the principles of organic agriculture and this percentage is far higher than the organic cereal share of 0.6% in the world. Besides having one of the largest adoption rates of organic agriculture, Switzerland is an interesting model to study how management, driven by political and economic decisions, as well as contrasting environments influence wheat performance. The widespread adoption of contrasting production systems and pronounced differences in environmental conditions that stem from a large landscape heterogeneity renders substantial variation in limiting factors (water and nutrients), inputs (fertilizers and fungicides), and outputs (grain and protein yield). In Switzerland, as in many other countries, wheat productivity increased during the second half of the 20^th^ century through the deployment of improved genotypes with high yield potential, enhanced tolerance to diseases and pests, and the use of mineral fertilizers and pesticides. Breeding wheat for high performance had raised wheat productivity dramatically during this period. The introduction of shorter genotypes allowed higher levels of nitrogen (N) application as well as later applications, which, influence both productivity and quality (Cruppe et al., 2017; Corassa et al., 2018). However, the achievements in productivity were accomplished to some extent at the cost of losing soil organic matter and other ecosystem services and polluting the environment (Paustian et al., 2016). To remediate these undesired effects, governments have introduced limitations for the use of certain inputs (e.g. mineral fertilizers and nocuous pesticides). In Switzerland, agricultural policy has introduced incentives for producing crops with fewer inputs or directly without some of them, as it is the case of organic agriculture with synthetic pesticides and mineral fertilizers (Lehmann and Stucki, 1997; Finger and Lehmann, 2012; Mascher and Willi, 2018). Production systems that are highly productive, resilient to changes in climate and minimize environmental harm are critically needed. A look into the genetic progress under different production systems, particularly organic ones, could be essential to identify opportunities to close gaps in productivity and in objectives (productivity vs environmental soundness).

Because the benefits of specific managements may depend on the genotype, variety trials in many countries are conducted under different cropping systems. Fischer (2009) estimated that approximately 0.6% of the 1.1% annual wheat yield gain in Australia is from improved management and 0.5% is from improved genotypes and G × management interactions. Cooper et al. (2001) examined the magnitude of G × management × environment interactions for grain yield and grain N concentration in multi-environment trials involving 272 advanced breeding lines and reported that the G × management component of this three-way interaction was the largest source of variation for both grain yield and grain N concentration. These findings not only indicate the importance of each component of the interaction to achieving high yields but also the potential to exploit such interactions to maximize grain yield and quality. However, wheat varieties registered specifically for organic agriculture rarely outperform genotypes that were bred for conventional management when both are tested under organic conditions (Przystalski et al., 2008). This has been attributed to the fact that breeding and cultivar registration for conventional management is conducted throughout several years under a broad range of environments, including treatments where pests and diseases are not controlled. Although the experimental design of most official-variety-trials do not allow quantifying G × management interactions, they can be used to quantify the relative importance of other factors (e.g. environments and genotypes) and inspect genetic progress and grain yield stability. Genotypes by Environments interaction (G × E) leads to variance differences and rank changes among genotypes (Crossa et al., 2004) and prevent higher levels of productivity and quality from being achieved (Jarquín et al., 2014). A better understanding of the impact of this interaction for different production systems may shed light on whether a breeding strategy for broad or narrow adaptability is more suitable given the production system.

The global demand for wheat is expected to rise driven by population and income growth (Charles et al., 2014). Besides a high productivity to respond to an increasing food demand, temporal and geographical stability of production will become a great challenge under a changing and less predictable climate (Schmidhuber and Tubiello, 2007). However, studies about production systems are generally focused on productivity while less attention is given to yield stability (Macholdt and Honermeier, 2017). Yet, farmers seek to reduce year-to-year variability in productivity to minimize income fluctuations. A recent study shows that the resilience of European wheat under conventional high-inputs has been declining because modern varieties have a reduced capacity as compared to older ones to respond to climatic variability and anomalies (Kahiluoto et al., 2019). Loss of resilience under a more unpredictable climate would represent a serious risk for the forthcoming future (Wójcik-Gront, 2018). Most analyses of production systems have focused on relatively short-term experiments and/or on single or few genotypes. However, studies that cover a period of 10 years or more are scarce (Kleinman et al., 2018). Furthermore, interactions between cropping systems and genotypes are well documented (e.g. Cober and Morrison, 2015) and the use of one genotype may create a bias in favor of one production system (Büchi et al., 2016).

This study analyses genotypes × environments interaction (G × E), genetic progress, and grain yield stability under contrasting production systems. We analyzed datasets obtained from three Swiss trial-networks of winter wheat designed to evaluate genotypes under organic, conventional low-inputs (150 kg N ha^−1^ with no fungicide application) and conventional high-inputs (170 kg N ha^−1^ with fungicide application) production systems.

## Materials and Methods

### Data Sources

**Figure 1:**
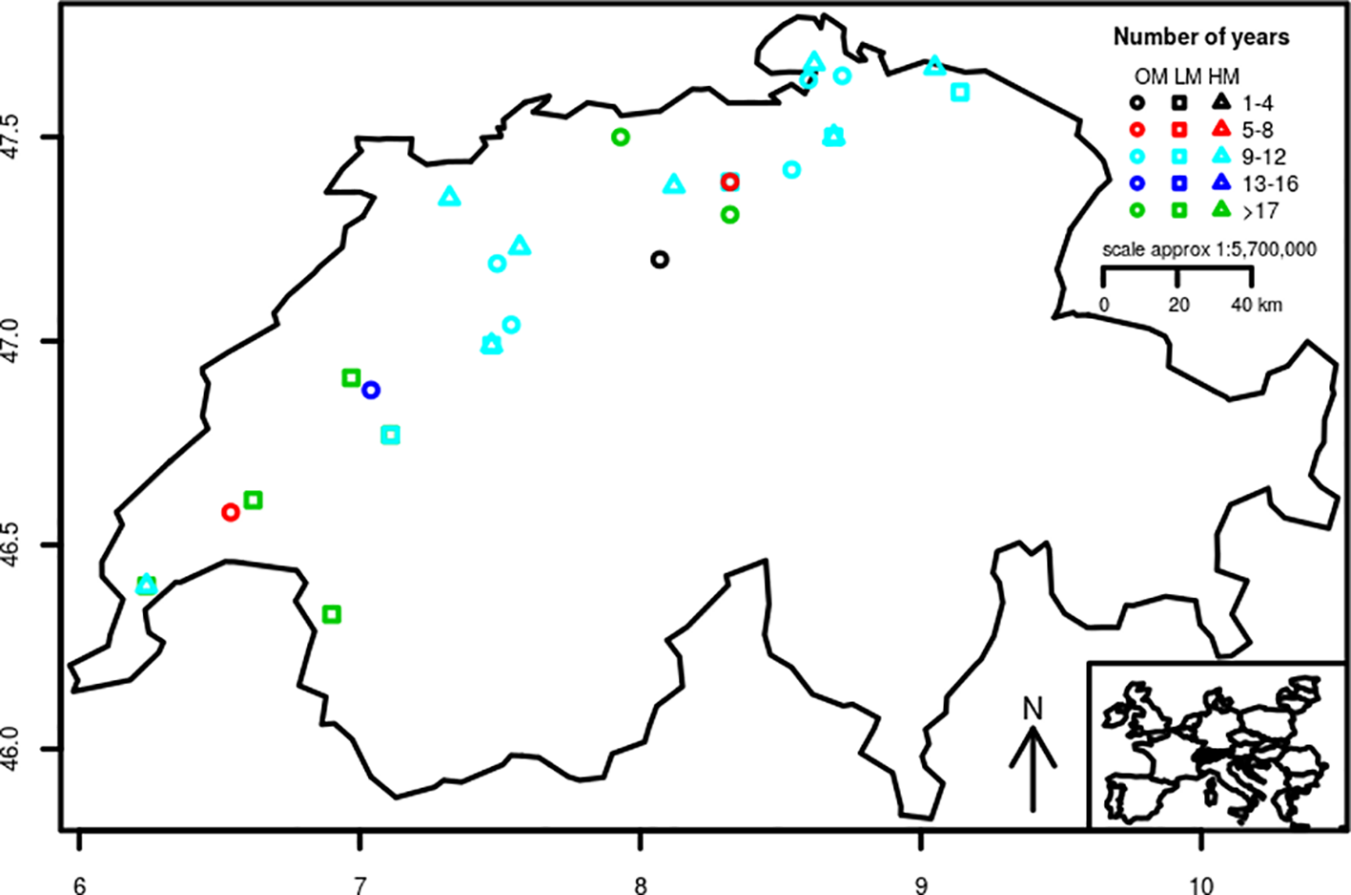
Distribution across Switzerland of the sites of the conventional low-inputs (LM), conventional high-inputs (HM), and organic (OM) trial networks of winter wheat varieties. Colors show the number of years that experiments were repeated at specific sites.

The datasets used in this study were obtained from three trial-networks of winter wheat varieties designed to evaluate genotypes in the context of three different production systems. The production systems were conventional management with low-inputs (LM), conventional management with high-inputs (HM), and organic management (OM). Besides the production system, the three networks differed in the locations where the experiments were conducted (**Table 1**) and the germplasm evaluated. The experimental sites were distributed across the wheat main production area of Switzerland (**Figure 1**). They were situated between 376 and 707 m a.s.l. and on soils that are mostly classified as Cambisols (World Resource Base, FAO) except for the site in Vouvry (**Table 1**) whose soil is classified as Fluvisol. Soil pH and organic matter content ranged between 6.7 and 8.1 and between 15 and 33 g kg^−1^, respectively. Field trials within each site were arranged as Lattice (conventional management with low-inputs), Latin square (conventional management with high-inputs), and randomized complete block (organic management) designs and always with three replications. The experimental plots covered 7.1 m^2^ (4.75 × 1.50 m) and consisted of eight rows with an inter-row distance of 0.16 m. Plots were separated by 1.3 m and sown at a rate of 350 (conventional management) and 380 seeds m^−2^ (organic management). Sowing and harvest took place each year during the months of October and June–July, respectively.

**Table 1:** Main characteristics of the sites of the conventional low-inputs (LM), conventional high-inputs (HM), and organic (OM) trial networks of winter wheat varieties.

Site	Alt.^a^	Prec.^b^ (mm)	Evap.^c^ (mm)	Evaluation networks in the site	Time Span	Grain Yield (dt ha^−1^)^d^		
						Organic	Conv. low-inputs	Conv. high-inputs
Assens	707	1,107	778	LM	1998–2018	NI^e^	72.27 ± 0.36	NI
Avenches	480	838	697	OM	2005–2018	39.15 ± 0.51	NI	NI
Bünzen	441	1,356	491	OM	1998–2018	56.50 ± 0.36	NI	NI
Changins	376	971	760	LM,HM	1998–2018, 2008–2018	NI	68.17 ± 0.30	68.57 ± 0.46
Courtemelon	441	897	673	HM	2008–2018	NI	NI	78.68 ± 0.58
Dickihof	416	848	612	OM	2008–2018	46.81 ± 0.48	NI	NI
Ellighausen	537	1,030	647	LM	1998–2008	NI	72.57 ± 0.31	NI
Grangeneuve	620	898	475	LM,HM	1998–2018, 2008–2018	NI	70.86 ± 0.36	79.73 ± 0.49
Hindelbank	519	1,018	541	OM	1999–2018	51.09 ± 0.36	NI	NI
Knutwil	541	1,182	530	OM	1997–2009	50.56 ± 0.43	NI	NI
Liebegg	510	1,679	651	HM	2008–2018	NI	NI	73.12 ± 0.43
Lindau	449	1,267	669	LM,HM	2008–2018, 2008–2018	NI	74.46 ± 0.39	77.47 ± 0.50
Moudon	540	953	671	LM,HM	1998–2018, 2008–2018	NI	72.26 ± 0.27	77.19 ± 0.47
Nennigkofen	456	1,031	528	OM	2008–2018	47.65 ± 0.37	NI	NI
Neuhausen	461	850	614	HM	2008–2018	NI	NI	73.68 ± 0.46
Portalban	529	808	691	LM	1998–2018	NI	75.34 ± 0.36	NI
Rheinau	400	854	618	OM	2001–2013	34.94 ± 0.33	NI	NI
Riedholz	471	1,031	528	HM	2008–2018	NI	NI	74.71 ± 0.50
Salenstein	400	844	608	HM	2008–2018	NI	NI	71.32 ± 0.49
Seebach	420	1,037	668	OM	2008–2018	51.50 ± 0.47	NI	NI
Sulz bei Künten	420	1,356	491	LM,OM	2008–2018, 2002–2007	50.38 ± 0.50	54.70 ± 0.39	NI
Vufflens	478	1,107	778	OM	2010–2018	41.98 ± 0.46	NI	NI
Vouvry	382	966	735	LM	1998–2018	NI	64.65 ± 0.33	NI
Wegenstetten	441	1,063	720	OM	1999–2018	44.95 ± 0.35	NI	NI
Zollikofen	564	1,018	541	LM,HM	2008–2018, 2008–2018	NI	78.68 ± 0.43	85.19 ± 0.49

^a^ Alt., altitude is expressed in m above sea level; ^b^Prec., average annual precipitation, ^c^Evap., average annual evapotranspiration, ^d^Data are means across years ± standard error of the mean; ^e^NI is site not included in the network.

In addition, we considered data from the Food and Agriculture Organization of the United Nations (FAO, 2019) to compare the results obtained in the variety trials with those that summarize the evolution and general characteristics of wheat productivity in Switzerland. FAO grain yield data are indirectly estimated from national production quantity and total area harvested and we used it here because long-term farm-level data were not available.

### Germplasm in the Swiss Winter Wheat Trial-Networks

The genotypes included released varieties, advanced breeding lines, lines submitted for registration or post-registration in Switzerland. According to the information provided by the seed companies, the vast majority of the genotypes were grown under conventional management during the selection stages of the breeding program. Selection was performed under diverse environments and across many years that exposed genotypes to variable climatic conditions. The set of genotypes also included varieties declared to have been bred and/or to be suitable for organic production.

Overall, the study considers results from 300 (conventional low-inputs), 81 (conventional high-inputs), and 102 (organic) genotypes in total. For the analyses of variance components (of grain yield and N concentration), yield trends, and stability, we considered only genotypes that were included at least three years in one of the trial-networks. By this, our analyses base on genotypes that fulfill the requirements to be registered in Switzerland. These genotypes may be assumed as genotypes that farmers would grow in their farms. Finally, the subset for the latter analyses included 58 (conventional low-inputs), 35 (conventional high-inputs), and 43 (organic) genotypes.

### Growing Conditions

Trials under conventional management were conducted on research stations of public institutes or educational centers while trials under organic management were mostly conducted in organic farms by public research institutes. Trials under organic management were conducted under the principles of organic agriculture, namely without using synthetic inputs (i.e., no synthetic pesticides and no mineral fertilizers). Wheat was fertilized at a rate of 150 kg N ha^−1^ (conventional low-inputs), 170 kg N ha^−1^ (conventional high-inputs), and at approximately 90 kg N ha^−1^ (organic). The source of N was liquid manure for the organic management and inorganic N fertilizer split into two to three applications depending on year and site for the conventional management. Available P and K values ranged from 38 to 184 and from 99 to 318 kg ha^−1^, respectively. Fertilization with P or K was not regularly done, except when their concentration was below the recommended soil availability. Fungicides and growth regulators were systematically applied under conventional high-inputs while no disease control or growth regulators were applied under conventional low-inputs and organic management.

### Characterization of Sites

We used two statistics to characterize sites: i) repeatability (also known as single plot heritability) which provides information on how consistent is the performance of the genotypes in one site, and ii) the ability of the sites to differentiate genotypes (differentiability, hereafter).

We estimated repeatability with the following equation:

(1) $$r_{jk}=\frac{s_{g}^{2}}{s_{g}^{2}+s_{e}^{2}}$$

where *r_jk_* is the repeatability in year *j* at site *k* and *s^2^_g_* is the genotypic variance and *s^2^_e_* the error variance. We estimated differentiability following Utz (1973):

(2) $$d_{k}=1+\frac{\sum_{i}(X_{ik}-{\bar{X}}_{i.}-\;{\bar{X}}_{.k}+\;{\bar{X}}_{\mathrm{..}})({\bar{X}}_{i.}-{\bar{X}}_{\mathrm{..}})}{\sum_{k}{\left({\bar{X}}_{i.}-\;{\bar{X}}_{\mathrm{..}}\right)}^{2}}$$

where *d_k_* is the differentiability at site *k* and X are mean values obtained across genotypes $({\bar{X}}_{i.}),$ sites $\left({\bar{X}}_{.k}\right)$ or both (*X*_*ik*_) and ${\bar{X}}_{\mathrm{..}}$is the general mean.

### Variance Components Analyses

We used the following model to quantify the amount of variance explained in grain yield and N concentration by specific factors:

(3) $$t_{ijk}=\;\mu +\;v_{i}+s_{k}\;+\;y_{j}+\;vs_{ik}+\;vy_{ij}+\;sy_{jk}+vys_{ijk}+\;e$$

where *t_ijk_* is the response variable (i.e. grain yield or grain N concentration) of genotype *i* in year *j* at site *k*; *µ* is the trial series mean; *v_i_* is the effect of genotype *i*; *s_k_* is the effect of site *k*; *y* is the effect of year *j; vs_ik_* is the interaction of variety *i* in site *k*; *vy_ij_* is the interaction of variety *i* in year *j*; *sy_jk_* is the interaction of site *k* with year *j*; *vys_ijk_* is the three-way interaction among variety *i*, site *k* and year *j*; and *e* is a residual comprising variation unexplained by the previous components. The model was fitted using maximum likelihood (ML) as implemented in the R (R Core Team, 2015) package “lme4” (Bates et al., 2015).

### Analyses of Trends in Grain Yield

We evaluated five statistical models (i.e. linear, quadratic, linear piecewise, logistic, and asymptotic) for their performance fitting the relationship between grain yield data from FAO and years (Grassini et al., 2013). The models were used as defined in the R packages “easynls” (i.e. quadratic), “segmented” (i.e. linear piecewise), and “stats” (i.e. logistic and asymptotic). Selection of the most suitable model was based on the AIC (Akaike information criterion) estimator (Akaike, 1998). The R function used for the linear piecewise model, estimates one or more breakpoints based on the slope parameters and changes in the linear relation. Long-term yield trends have genetic and non-genetic components which can be differentiated by a linear mixed model (Friesen et al., 2016) with regression terms (Piepho et al., 2014). Improved agronomic practices and environmental changes account for the non-genetic components while the genetic component allows characterizing the impact of breeding efforts on the productivity of released genotypes.

To account for both genetic and non-genetic effects on yield trends, we applied here the approach developed by Mackay et al. (2011) and extended by Piepho et al. (2014). The following model was fitted using restricted maximum likelihood (REML) as implemented in the R package “lme4”:

(4) $$g_{ijk}=\;\mu +\;v_{i}+\;s_{k}\;+\;y_{j}\;+vs_{ik}+vy_{ij}+sy_{jk}+\;e\;$$

where *g_ijk_* is the grain yield of genotype *i* in year *j* at site *k*; *µ* is the mean; *v_i_* is the effect of genotype *i*; *s_k_* is the effect of site *k*; *y* is the effect of year *j*; *vs_ik_* is the interaction of variety *i* with site *k*; *vy_ij_* is the interaction of variety *i* with year *j*; *sy_jk_* is the interaction of site *k* with year *j*; and *e_ijk_* is a residual comprising both genotype × site × year interaction as well as the error of the mean. To account for genetic effects on yield trends, we explicitly incorporated regression terms to model the genetic source in the grain yield trend by using the year that each genotype entered the trials for the first time:

(5) $$G_{i}=\beta y_{0i}+H_{i}$$

where β is a fixed regression coefficient for genetic trend, y_0i_ is the first year the genotype *i* entered the trials, and *H_i_* models a random normal deviation of G_i_ from the genetic trend line.

Despite some apparent non-linear relations, in the cases where visual assessments suggested potential deviations from a linear relationship, we did not find a non-linear substitute. Therefore, we report linear regression results. These models assume that at least some sites are used across several years. All effects except *µ*, *v_i_*, *y_j__,_*, and *y*_0_ *_i_* are assumed to be random with constant variance. We estimated adjusted means with the R package “emmeans”.

Some authors proposed to add regression terms to account for breakdown of disease resistance (Mackay et al., 2011; Laidig et al., 2017). Although we made such attempts, they did not improve the performance of the models. We attribute this to two reasons: i) in reality individual genotypes succumb to disease abruptly and often at non-linear rates (Mackay et al., 2011) and ii) the genotypes stayed on average for short times in the trials (Perronne et al., 2017). The average age of the genotypes was 4.57, 4.32, and 2.94 years under organic, conventional low-inputs, and conventional high-inputs, respectively.

### Grain Yield Stability and Interannual Variability

Grain yield stability can be measured in different ways (Lin et al., 1986). One way to measure grain yield stability that was often used for comparing cropping systems is the coefficient of variation (CV) (e.g. Knapp and Van der Heijden, 2018), which divides the variability in grain yield (expressed as standard deviation) by the grain yield mean. The advantage of this approach is that it provides a measure of variability corrected by the level of grain yield achieved. Different approaches were also followed to study the interannual variability on the grain yield of crops. Here we followed the approach of Penalba et al. (2007) that considered the absolute values of the difference between the mean grain yield of one year and the mean grain yield of all the years throughout the duration of the study. Since genetic progress may increase grain yield with time and show deviations from the mean grain yield of all the years that are not associate to climate, we divided the aforementioned difference by the mean of the year and express this parameter as a ratio. We refer to this parameter as interannual deviation ratio. The rationale of these analyses was to determine if there were reductions in grain yield stability and increases in interannual variability due to climate change. We additionally wanted to determine if there were differences in these parameters among production systems. We used Mann-Kendall and Hartley’s tests in these analyses.

### Statistics and Presentation of Results

We used R (R Core Team, 2015) for all statistical analyses. Performance at the sites were evaluated using the following indicators: CV (data not shown), repeatability (**Table 2**), differentiability (**Table 2**), and the visual inspection of heat maps of grain yield for spatial patterns associated to gradients in soil fertility or another factor that may disrupt the real differences among the tested genotypes (data not shown). Repeatability, differentiability and CV were calculated per year but displayed as means across years in order to summarize results. We conducted a Hartley’s test as implemented in the R’s package “stats” to assess differences in variances of grain yield among production systems and a Mann–Kendall test as implemented in the R’s package Kendall to determine if there were time series trends within production systems in the CV and interannual deviation ratios of grain yield.

**Table 2:** Average repeatability (m.r.) and average ability to differentiate genotypes (m.d.) of the sites included in the study.

Site	Organic			Conventional Low-inputs			Conventional High-inputs		
	m.r. ± s.e.^a^	m.d. ± s.e.	n^b^	m.r. ± s.e.	m.d. ± s.e.	n	m.r. ± s.e.	m.d. ± s.e	n
Assens		NI^c^		0.87 ± 0.02	1.12 ± 0.11	17		NI	
Avenches	0.36 ± 0.12	0.99 ± 0.25	12		NI			NI	
Bünzen	0.60 ± 0.08	1.11 ± 0.19	20		NI			NI	
Changins		NI		0.87 ± 0.05	0.99 ± 0.10	20	0.95 ± 0.01	1.00 ± 0.12	10
Courtemelon		NI			NI		0.92 ± 0.02	1.15 ± 0.14	10
Dickihof	0.30 ± 0.12	0.79 ± 0.31	10		NI			NI	
Ellighausen		NI		0.84 ± 0.04	0.92 ± 0.11	21		NI	
Grangeneuve		NI		0.82 ± 0.04	0.93 ± 0.11	21	0.88 ± 0.03	1.03 ± 0.13	10
Hindelbank	0.60 ± 0.08	1.01 ± 0.22	17		NI			NI	
Knutwil	0.50 ± 0.11	1.09 ± 0.16	11		NI			NI	
Liebegg		NI			NI		0.94 ± 0.01	0.91 ± 0.12	10
Lindau		NI		0.84 ± 0.05	1.09 ± 0.11	9	0.91 ± 0.02	1.04 ± 0.12	11
Moudon		NI		0.76 ± 0.05	1.01 ± 0.12	21	0.87 ± 0.03	0.99 ± 0.15	11
Nennigkofen	0.70 ± 0.09	1.22 ± 0.23	11		NI			NI	
Neuhausen		NI			NI		0.90 ± 0.02	0.92 ± 0.13	11
Portalban		NI		0.89 ± 0.01	1.13 ± 0.10	21		NI	
Rheinau	0.39 ± 0.08	0.53 ± 0.18	11		NI			NI	
Riedholz		NI			NI		0.95 ± 0.01	1.02 ± 0.10	11
Salenstein		NI			NI		0.94 ± 0.01	0.95 ± 0.10	10
Seebach	0.60 ± 0.11	1.11 ± 0.24	10		NI			NI	
Sulz bei Künten	0.44 ± 0.12	1.07 ± 0.17	6	0.73 ± 0.06	0.66 ± 0.12	10		NI	
Vouvry		NI		0.92 ± 0.01	1.06 ± 0.13	17		NI	
Vufflens	0.39 ± 0.12	1.17 ±0 .27	7		NI			NI	
Wegenstetten	0.71 ± 0.07	0.96 ± 0.18	19		NI			NI	
Zollikofen		NI		0.86 ± 0.02	1.01 ± 0.10	11	0.93 ± 0.01	0.99 ± 0.12	10

^a^s.e. is standard error of the mean; ^b^n is the number of years used in the calculations; ^c^NI is site not included in the network. The parameter was estimated from grain yield under conventional low-inputs (LM), conventional high-inputs (HM), and organic (OM). trial networks.

## Results

### Summary Statistics and Characteristics of Sites

Averages across sites and years (**Table 1**) show, as expected, that grain yield was the highest in the network where wheat genotypes were grown under conventional practices with high-inputs (75.97 dt ha^−1^). They were followed by those where the genotypes were grown under conventional practices with low-inputs (70.40 dt ha^−1^). The genotypes grown under organic practices showed the lowest grain yield mean (46.86 dt ha^−1^). These results indicate a yield difference of 5.57 and 29.11 dt ha^−1^ for the conventional low-inputs and organic production systems, with respect to the conventional management with high-inputs. The corresponding values considering only the grain yields observed the last three years with common genotypes in the compared networks are 14.32 and 20.23 dt ha^−1^, and in both cases these differences are statistically significant (p <0.05). A comparison for both common genotypes and sites was only possible between conventional low-inputs and conventional high-inputs and it showed a difference of 11.35 dt ha^−1^.

The CV was systematically higher under organic management, where only one site had a CV <10%. Overall, the CV was higher when the amount of inputs used was lower (organic > conventional low-inputs > conventional high-inputs) and showed always values below 10% under conventional managements. In contrast, repeatability showed the opposite ranking; values were higher when the amount of inputs used was higher (organic < conventional low-inputs < conventional high-inputs). Thus, the evaluation of genotypes tended to be more consistent across years in the sites under conventional high-inputs than low-inputs. Under organic management, there were sites with very low (i.e. Assens, Dickihof) and low (i.e. Rheinau, Sulz bei Künten, and Vufflens) repeatability, showing that evaluations in these sites tended to be more inconsistent across years than those conducted at other sites. Despite the limitations in the organic network, the sites of Bünzen and Nennigkofen show a high ability to differentiate genotypes (values >1). Similarly, the sites of Assens, Portalban, Lindau, and Vouvry under conventional low-inputs and Courtemelon and Lindau under conventional high-inputs showed an ability above the average to differentiate genotypes. In short, higher levels of inputs resulted in less variable grain yield (lower CV), higher consistency across years (repeatability), and higher ability to differentiate genotypes (differentiability).

### Variance Components Analyses

**Figure 2:**
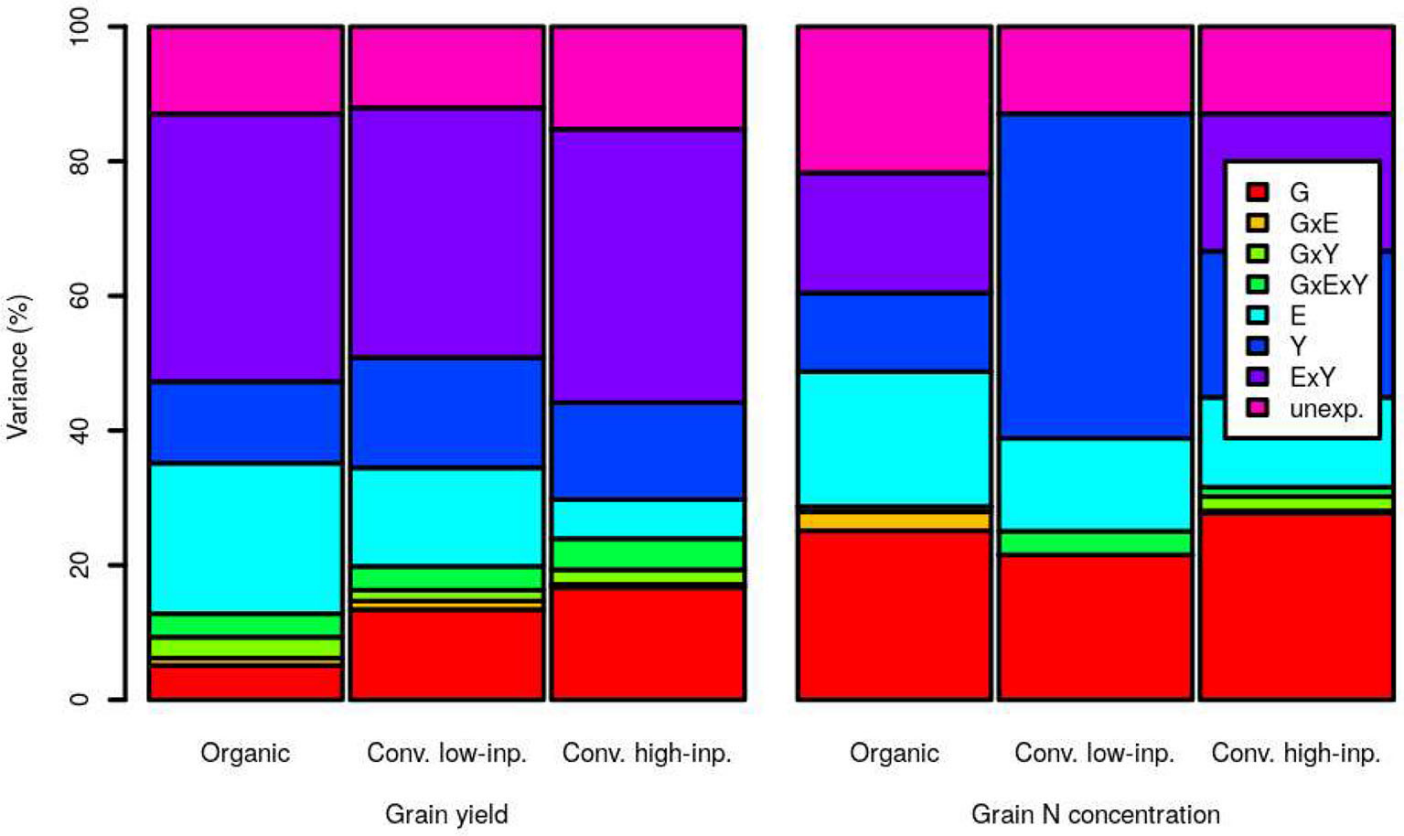
Variance component analyses for grain yield and grain nitrogen (N) concentration within an organic (1998–2018), conventional low-inputs (Conv. low inp.) (1998–2018) and conventional high-inputs (Conv. high inp.) (2008–2018) variety-testing networks. Components considered were genotypes (G), environments (E), years (Y), the interaction genotypes by environments (G × E), the interaction genotypes by years (G × Y), the interaction environments by years (G × Y), and the interaction genotypes by environments by years (G × E × Y). We also show the amount of variance that remained unexplained by the models (unexp.).

**Figure 2** shows the results of variance components analyses for grain yield and N concentration in the three trial-networks. Five, 13 and 17% of the variance on grain yield was ascribed to genotypes effects under organic, conventional low-inputs and conventional high-inputs, respectively. The corresponding values were 13, 20, and 24% when taking into account also other components where the factor genotypes was involved through interactions. The factor genotypes explained a higher proportion of the variance in grain N concentration than in grain yield, with percentages of 25, 22, and 28 in the organic, conventional low-inputs and conventional high-inputs trial-networks, respectively. The corresponding values were 29, 25, and 32% when taking into account also other components where the factor genotypes was involved through interactions.

For grain yield, the factor that explained the highest proportion of variance was the environment by years interaction and the value of this proportion was similar for the three production systems. Interestingly, the amount of variance explained by the environment as a single factor diminished as the inputs level was higher (organic > conventional with low-inputs > conventional with high-inputs). This reduction is almost equivalent to the increase in the proportion explained by genotypes alone or through interactions with increasing use of inputs (conventional with high-inputs > conventional with low-inputs > organic).

For grain N concentration, the main difference among production systems is the amount explained by years as a single factor, which was surprisingly high under conventional low-inputs. In any case, the proportion of variance explained by years was also higher for grain N concentration than for grain yield in the other two cropping systems. Interactions among factors explained a lower amount of the total variance in grain N concentration than in grain yield.

### Grain Yield Trends

**Figure 3:**
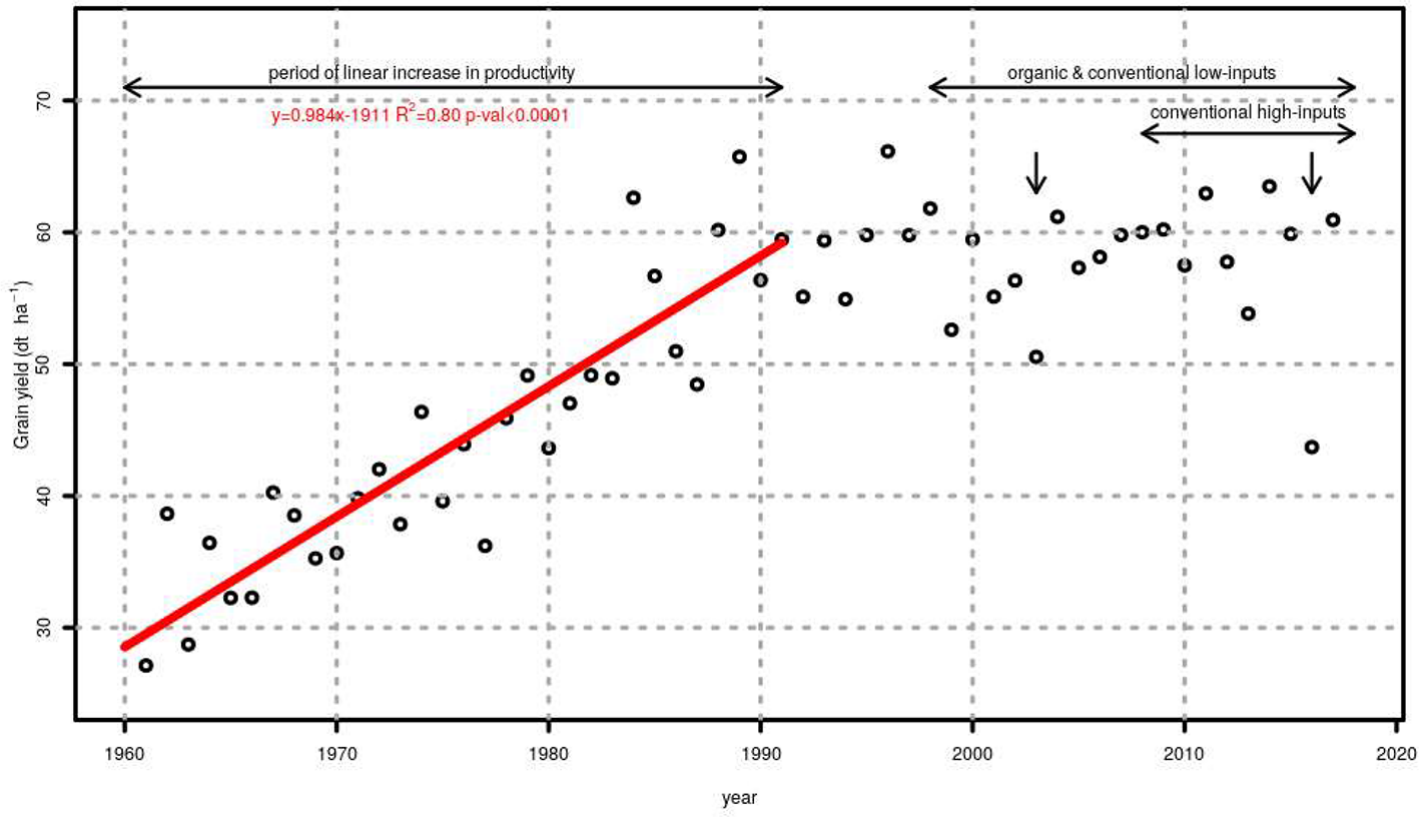
Average grain yield of winter wheat in Switzerland between 1961 and 2017. A linear trend summarizes the evolution from 1961 to 1989 (red line). The period during which genotypes were tested under organic, conventional low-inputs and conventional high-inputs is shown using horizontal arrows while vertical arrows show years with exceptionally severe droughts.

**Figure 3** shows the average grain yield of winter wheat in Switzerland between 1961 and 2017. This figure also presents the period in which the genotype evaluations presented here took place. The linear piecewise model (AIC = 339.9) showed to be more suitable than linear (AIC = 373.9), quadratic (AIC = 427.5), logistic (AIC = 347.7), and asymptotic (AIC = 351.6) models for summarizing the relationship between grain yield and years. The breakpoint between the period of linear increase in productivity and the period of stagnating yields was estimated to be the year 1991. When only data between 1961 and 1991 was considered (line in **Figure 3**), the slope of the linear regression for the period of linear increase in productivity was 0.98 dt ha^−1^ y^−1^ (standard error of the mean 0.09 dt ha^−1^ y^−1^).

**Figure 4:**
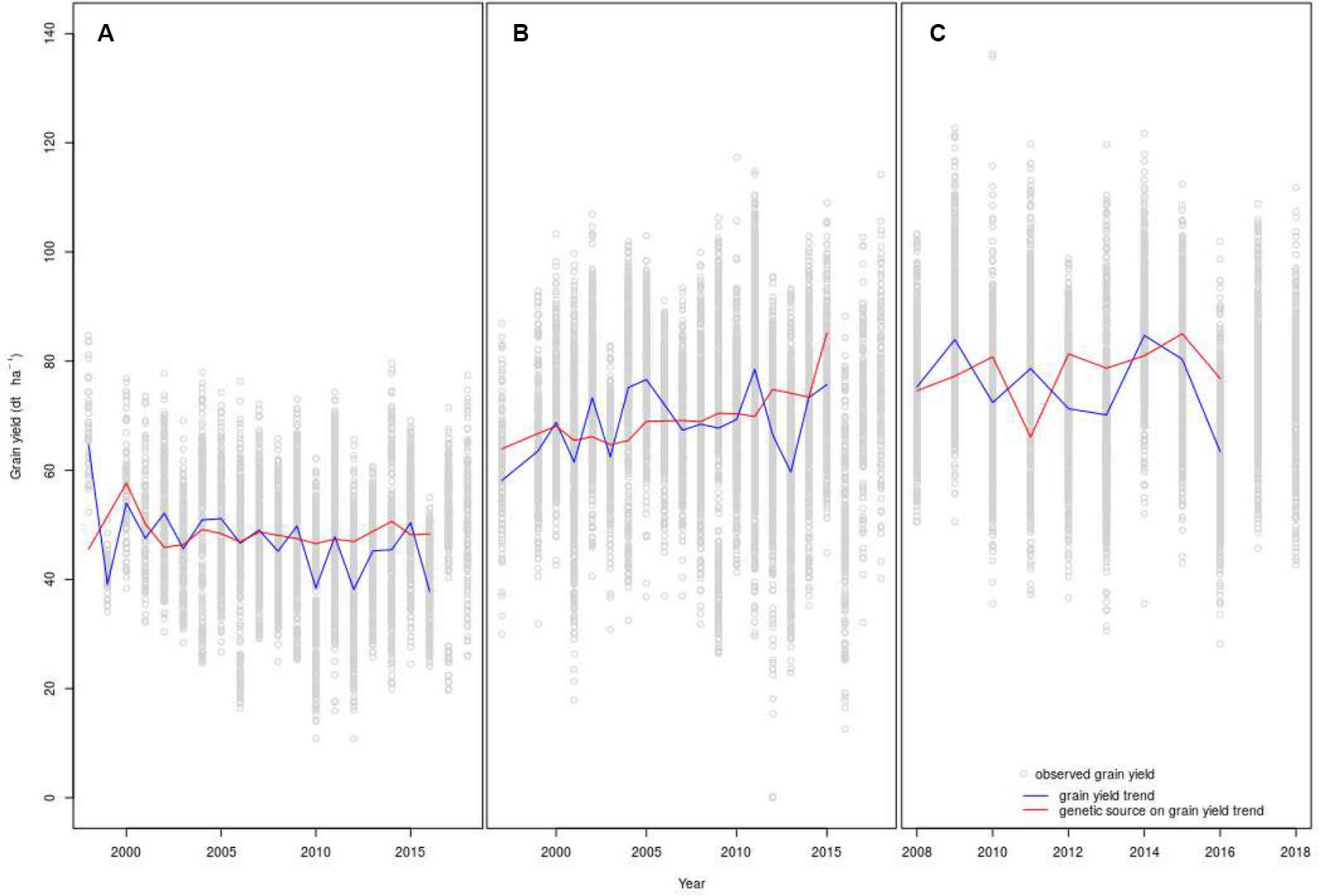
Grain yield trends and genetic sources on the grain yield trends under organic **(A)**, conventional low-inputs **(B)**, and conventional high-inputs **(C)** management. The fit on the models is shown until 2016 because the analysis was performed on genotypes that remained in the trials at least three years. Because of this criterion, the year 2016 was the last year available to consider the year that a genotype entered the variety trials. X-axis of panels **(A–C)** have different scales.

Points in **Figure 4** show grain yields of genotypes from 1998 to 2018 (**Figure 4A**: organic system and **Figure 4B**: conventional system with low-inputs) and from 2008 to 2018 (**Figure 4C**: conventional with high-inputs). Fitting data with non-linear models did not show an advantage over linear ones. Thus, it was not necessary to replace linear regression models by non-linear ones (Lopes et al., 2012; Piepho et al., 2014). Grain yield trends and the genetic source of grain yield trends are plotted with lines. The slope of the grain yield trends observed under conventional low-inputs was not different from zero (**Table 3**) while the corresponding slope for the conventional high-inputs and organic managements were negative. However, only under organic management this slope was significantly negative. In contrast, the genetic source of the grain yield trend was positive in the three studied networks (**Table 3**) and it was significant and marginally (p < 0.10) significant for the conventional low-inputs and conventional high-inputs managements, respectively. While the increase in the genetic source of grain yield was almost null under organic management, genetic effects increased steadily under conventional managements. This increase represented 0.58 and 0.68 dt ha^−1^ y^−1^ with low- and high-inputs, respectively. The estimated genetic contribution to the grain yield trend under conventional low-inputs (0.58 dt ha^−1^ y^−1^) is in the same range of the value obtained from FAO data for the period 1961-2015 (0.58 dt ha^−1^ y^−1^). In both conventional production systems, the contribution of genetics to the grain yield was often below the average before 2007, while after this year it was above the average in 8 out of 9 and in 5 out of 9 years with low- and high- inputs, respectively (**Figures 4B**, **C**). In the conventional systems, independently of the levels of inputs, the contribution of genetics to the grain yield trend was above the average in 2015, a year with a severe drought in Switzerland. This was also the case under conventional low-inputs in 2003, another year with a severe drought. In contrast, under organic management the genetic component of the grain yield trend was below the average in 2003 and 2015.

**Table 3:** Regression parameters used in mixed linear models of grain yield of winter wheat to estimate the slopes for grain. yield trends and genetic sources on grain yield trends.

Trial Network	Grain yield trend (dt ha^−1^ y^−1^)		Genetic source on grain yield trend (dt ha^−1^ y^−1^)	
	Estimate ± s.e.^a^	Pr(>|t|)	Estimate ± s.e.	Pr(>|t|)
Organic (OM)	−0.35 ± 0.17	0.03*	0.09 ± 0.08	0.32ns
Conventional with low-inputs (LM)	0.16 ± 0.16	0.33ns	0.58 ± 0.13	<0.0001***
Conventional with high-inputs (HM)	−0.41 ± 0.34	0.23ns	0.68 ± 0.35	0.06†

a s.e. is standard error of the mean; ns is not significant at the 0.10 probability level; †, *, *** are significant at the 0.10,0.05, and 0.001 probability levels, respectively.

### Grain Yield Stability and Variability

**Figure 5:**
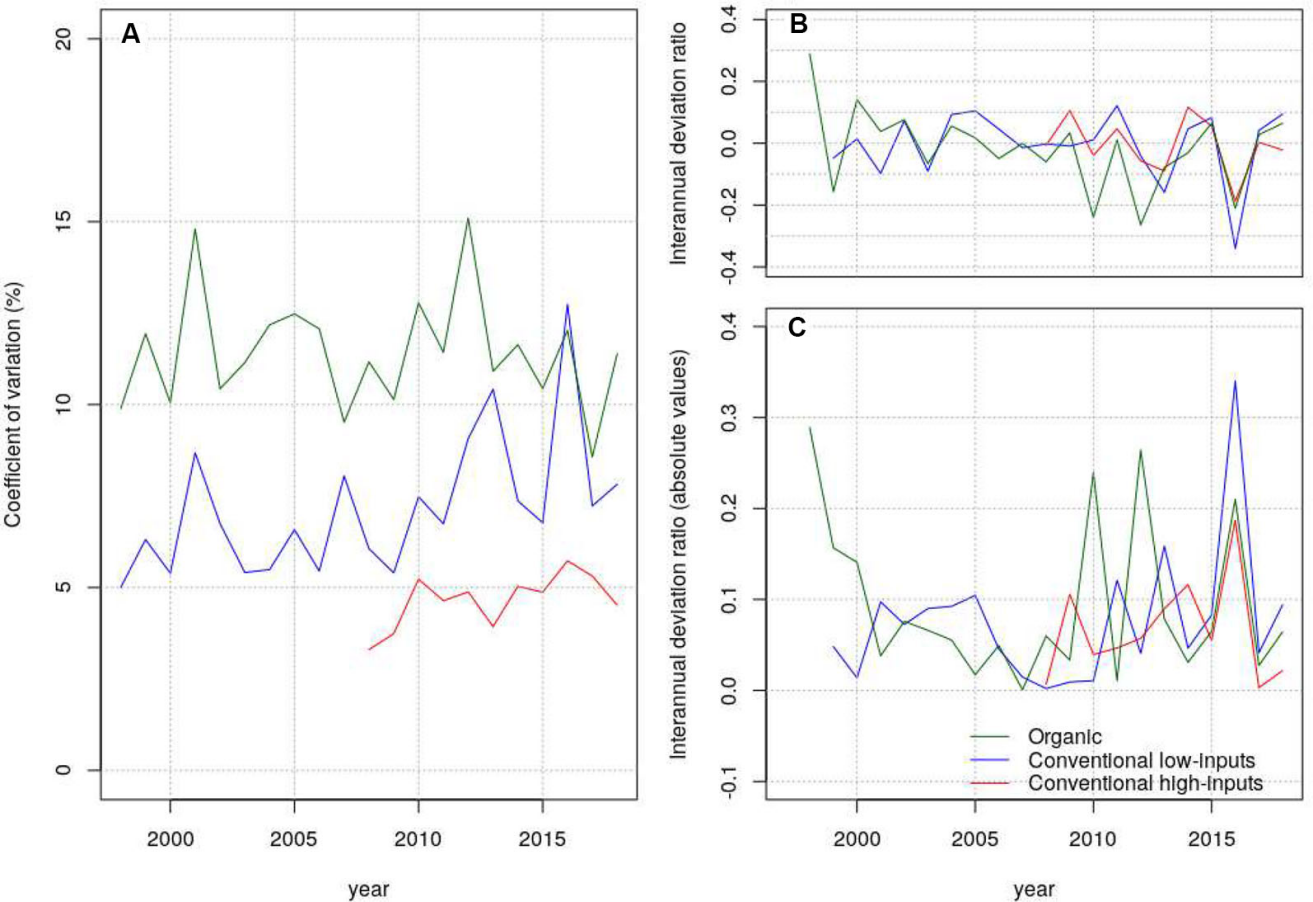
Coefficient of variation **(A)**, interannual deviation ratio **(B)** and absolute values of the interannual deviation ratio **(C)** estimated from grain yield under contrasting production systems.

We inspected the yield-stability considering the CV of all the genotypes that stayed in the trial-networks for more than two years (**Figure 5A**). The average values of CV were 18.5, 16.5, and 14.0% under organic management, conventional low-inputs, and conventional high-inputs, respectively. Thus, it was systematically higher under organic than under conventional management. A Hartley’s test to assess differences between variances showed marginally significant differences between conventional low-inputs vs conventional-high inputs and significant differences between conventional high-inputs and organic. We were particularly interested to know if there was a trend towards a decrease in stability (or increase in interannual variability) triggered by climate change and if there were different patterns among production systems. We tested this hypothesis in different ways. A Mann–Kendall test on the CV of grain yield through time revealed only a marginally significant trend under organic management. We considered the interannual-deviation-ratio of grain yield (**Figure 5B**) which showed positive and negative values (**Figure 5B**). Thus, it can be ruled out that deviations are due to grain yield increases from genetic progress. The consideration of absolute values for the interannual-deviation-ratio of grain yield suggests an increasing interannual variability (**Figure 5C**) also visible for the CV of grain yield under conventional low-inputs (**Figure 5A**). The onset of this increasing variability seem to be from 2007 onwards. However, the statistical tests that we considered did not reveal significant trends.

## Discussion

Grain yield and wheat quality are subject to unexpected outcomes from interactions between genotypes and environmental factors. This is a challenge in breeding and genotype evaluation programs (Cullis et al., 2000; Munaro et al., 2014). Among the most important environmental factors that modify genotypic performance are soil characteristics, N availability, rainfall, and temperatures during ripening. Numerous studies show the influence of G × E on wheat’s grain (e.g. Bilgin et al., 2016) and protein (Finlay et al., 2007; Williams et al., 2008) yields. Grain yield was highly influenced by environment by year interaction in the three production systems considered here (**Figure 2**). The realization of the yield potential of varieties relied on the use of inputs; when the levels of inputs were higher, the variance on grain yield explained by genotypes was higher. Hence, a higher use of inputs reduced the impact of environment on this trait. This suggests that with a lower level of inputs, selection programs may need to include a larger number of environments to detect stable performing genotypes. Breeding programs for wheat cannot neglect the importance of quality and the factor genotype influenced to a higher extent protein than grain yield. This may be explained by the fact that the genetically determined composition of gluten is the main determinant of genotypic differences in grain protein concentration (Payne et al., 1987; Jiang et al., 2019). The comparable contribution of genotypes to grain N concentration across production systems suggest that selection strategies for protein concentration may be less dependent on the type of production system considered. Overall, our results agree with general conclusions by other authors that grain yield and protein concentration were highly sensitive to environmental fluctuations (Bilgin et al., 2016; Laidig et al., 2017). Here we also show that this was the case for three contrasting production systems.

According to the FAO database, productivity of wheat in Switzerland has stagnated during the last 27 years. The fact that FAO grain yield data (**Figure 3**) are estimated from national production quantities and the total harvested area, may lead to misleading conclusions such as that grain yield stagnation may not really stem from an invariable yield potential of the winter wheat genotypes but just from changes in the area harvested. This is, however, unlikely because there were no major changes in the area harvested and grain yield measured directly in genotype trials also showed stagnation (**Figure 4** and **Table 3**) for different production systems. Under organic management, the yield trend was even negative with a significant slope (**Table 3**). The question if this stagnation in the productivity shows lack of genetic improvement in winter wheat will be addressed later, demonstrating that the answer depends on the production system considered. Among causes of stagnation in wheat productivity, different reasons have been suggested. They include lack of genetic improvement (Calderini and Slafer, 1998), changes in crop management (Brisson et al., 2010), worsening of environmental conditions caused by climate change (Hochman et al., 2017), use of low input levels (Patrignani et al., 2014), lack of crop rotations (Patrignani et al., 2014), soil degradation (Patrignani et al., 2014), as well as economic (Hafner, 2003), and political (Finger, 2010) factors. Political and economic decisions may lead to changes in agricultural practices such as a reduced use of inputs. In 1992, approximately at the onset of the period of stagnated productivity, subsidies to reduce the use of fungicides, insecticides, plant growth regulators, and synthetic stimulators were introduced in Switzerland. Finger (2008) propsosed also shifts towards production systems with lower inputs as one of the main reasons for the stagnation in wheat’s productivity. Another process that was observed in the last 10 years is that more farmers are targeting a market class with a higher quality requirement. The wheat varieties that they must grow to achieve these quality requirements have lower yield potential.

A possible pathway to break grain yield stagnation is through plant breeding. The contribution of breeding to increase grain yield in farms may depend however on the difference between current yields and potential yield in different regions and production systems (Battenfield et al., 2013; Patrignani et al., 2014). In the genotype trial networks, we found that yield stagnation is present independently of the production system (**Figure 4**) and the genetic component of the yield trends shows different outcomes depending on the production system considered (**Table 3**). While we found positive significant and marginally significant genetic effects on conventional low- and high- inputs, respectively, we did not detect evidence of significant genetic improvements under organic conditions. The procedure applied for conducting filial generations (F_2_ to F_5_) in a breeding program can affect the response of genotypes to management as shown for winter wheat for grazing vs. grain-only production systems (Thapa et al., 2010). A recent comprehensive study showed that breeding for high inputs enhances cultivar performance not only under high inputs but also in production systems with reduced agrochemical inputs (Voss-Fels et al., 2019). However, the study by Voss-Fels et al. (2019) did not include organic management. We did not find evidence that breeding for other production systems impacted genetic progress under organic management in the same extent; the genetic effects that we found under conventional management were not observed under organic management (**Table 3**). The parallel impact for conventional low- and high-inputs production systems was explained by the fact that most breeding programs expose their materials to conventional conditions of low and high inputs through the cycles of selection and testing (Voss-Fels et al., 2019). Besides the use of modern breeding techniques, continual gains in productivity and quality have been promoted by official genotype testing procedures that require trait improvement and consistency across diverse environments as a prerequisite for cultivar registration (Voss-Fels et al., 2019). Thus, traits that are important under high-inputs may be also relevant with lower inputs under the same production system and only the degree of expression of the trait may differ. In contrast, organic management may have differences in the sources and dynamics of nutrients (Lori et al., 2017) and in the spectrum of pest and diseases (Lammerts Van Bueren et al., 2011; Bilsborrow et al., 2013) compared to conventional management that may demand additional or different traits (Lammerts Van Bueren et al., 2011). An additional indication of the likely lower breeding effort for organic management is the higher average age of the varieties; fewer genotypes are submitted each year for the test under organic conditions, which results in a 55% higher age of the genotypes under organic management than conventional with high-inputs. Since the sites under organic management differed from those under conventional management, genotypes may in addition be less adapted to the sites where they have been tested. Such hypothesis is supported by the higher impact of the factor Environment, alone or interacting, under organic management than under conventional management (**Figure 2**). Finally, agronomic limitations that prevent the yield potential to be expressed under organic management cannot be fully ruled out as well as differences in breeding goals for conventional and organic production systems.

The most widely practiced and studied alternative to high-inputs agriculture is organic management. Studies of organic agriculture have revealed better performance than conventional practices on some parameters associated to sustainability but not all (Tuck et al., 2014). The concept of yield gap (Van Ittersum et al., 2013) allows identifying unlocked yield potential and it must be kept in mind that generally full yield gap closure is not economically feasible and not environmentally advisable. The yield gap for rainfed wheat was recently estimated for Switzerland at 3.7 t ha^−1^. This was based on an estimated yield potential for rain-fed conditions of 9.7 t ha^−1^ (Schils et al., 2018). This yield potential was determined with the WOFOST crop model considering temperature, day length, solar radiation, and genetic characteristics assuming absence of any water or other stress factors (Schils et al., 2018). The results obtained in the last three years (2015–2018) under conventional management with high-inputs shows an average performance of genotypes only 2 t ha^−1^ below the yield potential. Thus, the genotypes recently released may be reaching the biophysical potential of wheat in Switzerland. Besides the pressure from consumers and regulators to move towards production systems with fewer inputs, levering yield potential may be challenging for conventional management with high-inputs due to economic, environmental, and biophysical limitations. We estimated yield differences for organic and conventional management with low-inputs compared to conventional management with high-inputs. These differences can be considered a rough approximation because it compares results obtained in different sites, with different genotypes, and for experiments conducted throughout different periods. According to Laidig et al. (2017) it must be noted that grain yields on farms tend to be lower than in variety trials for three reasons: i) variety trials are usually ignored if they are not of sufficient quality; ii) the average age of varieties grown on-farm is usually higher than in variety trials; and iii) economic constraints imposed by the prices of inputs are usually considered on-farms but not on variety trials. Furthermore, genotype trials are generally located in the middle of fields and will not therefore suffer from the reduced grain yields at field margins from soil compaction and other limitations (Mackay et al., 2011). Although, organic agriculture originally addressed the demand of consumers for food free of synthetic pesticides and fertilizers, it has recently been proposed as a solution to revert the loss of soil organic matter and other ecosystem services (Gattinger et al., 2012). Different authors suggested that organic production systems can be a way to increase the sustainability of cereal production as long as it closes the yield gap with other production systems and meet the requirements necessary to sustain a growing world population (e.g. Trewavas, 2001). A meta-analysis by Ponisio et al. (2015) compared 1071 paired yield observations under conventional and organic agriculture and concluded that fields under organic management had on average 19.2% less yield compared to those under organic management. Cereal crops exhibited the greatest differences between organic and conventional systems, which the authors attributed to the extensive efforts since the Green Revolution for breeding high yielding varieties adapted to respond to high inputs. Early comparisons of organic and conventional systems suggested that the yield gap between organic and conventional production systems was going to decline over time (Smith et al., 2007). In the genotype trials evaluated here, which were mainly conducted in farms, there is no sign that the yield difference between organic and conventional practices declined during the studied period.

Agronomy may contribute significantly to reductions in yield gaps through a more optimized and efficient use of inputs (Chapagain and Good, 2015; Lollato et al., 2019). It might be useful to investigate explicitly and systematically how specific management practices (e.g. rotations, fertilization, and weed, diseases and pest control) could be altered in different production systems to maximize productivity and quality. The exploitation of interactions between genotype and crop management has, indeed, produced important changes in production strategies in the last century, shown for example by the use of shorter wheat varieties that can be fertilized with higher doses. Improved agronomy is necessary for production systems with reduced levels of inputs but also for those that rely on high inputs. The use of nitrogenous fertilizers presents a challenge, as the optimization of plant nutrition stabilizes yields and helps to reduce expansions in crop area but contribute substantially to the greenhouse gas emissions that promote climate change (Fowler et al., 2013).

Genotypes can contribute to attain a stable wheat production. The relative yield stability (i.e. stability assessed per unit yield produced) was the lowest with the least use of inputs. Among the differences in production systems, N availability may be a key element explaining the higher yield variation under organic management compared to conventional management with high inputs (Bilsborrow et al., 2013). Although, we observed an apparent increasing trend for CV with conventional low-inputs and for the interannual-deviation ratio, the trends were not statistically significant. This differs with other studies published (e.g. Kahiluoto et al., 2019) and we attribute this difference to the fact that we considered several genotypes instead of a few ones. The availability of adapted genotypes is therefore fundamental to achieve a stable wheat production in a given cropping system. The implementation of a strategy to use genotypes for climatic-risk mitigation will not however be easy without models that anticipate these risks or agronomic practices that rely on the use of genotypes mixtures.

## Data Availability Statement

Restrictions apply to the datasets: The datasets used in this study will be made available upon fulfilling certain requirements. However, they are not publicly available because a part of the data was obtained from a third party. Requests to use the organic, conventional high inputs and conventional low inputs datasets should be directed to LL (lilia.levy@agrscope.admin.ch).

## Author Contributions

LL, JH, and DP planned and designed the experiments. LL, JH, FM, DF and CB conducted the experiments and fieldwork. JMH analyzed the data. JMH, LL, FM, JH, DF, CB, RC, and DP wrote the manuscript.

## Funding

This study was supported by the project CerQual form the Swiss Federal Office for Agriculture.

## Conflict of Interest

The authors declare that the research was conducted in the absence of any commercial or financial relationships that could be construed as a potential conflict of interest.
